# The role of vitamin D in children with recurrent Tonsillopharyngitis

**DOI:** 10.1186/1824-7288-38-25

**Published:** 2012-06-08

**Authors:** Ismail Yildiz, Emin Unuvar, Umit Zeybek, Bahar Toptas, Canan Cacina, Sadık Toprak, Ayse Kilic, Salih Aydin

**Affiliations:** 1 Istanbul Medical Faculty, Department of Pediatrics, Istanbul University, Istanbul, Turkey; 2 Institute of Experimental Medical Researches, Department of Molecular Medicine, Istanbul University, Istanbul, Turkey; 3 Medical Faculty, Department of Forensics Medicine, Zonguldak Karaelmas University, Zonguldak, Turkey; 4 Istanbul Medical Faculty, Department of ENT Clinic, Istanbul University, Istanbul, Turkey; 5 Istanbul Tıp Fakultesi, Cocuk Klinigi, Capa, Istanbul, 34390, Turkey

**Keywords:** Child, Vitamin D, Infection, Tonsillitis

## Abstract

**Background:**

The exact etiology of recurrent tonsillopharyngitis in children is not clear. Recurrent tonsillitis in children has multifactorial etiology like most of the diseases in childhood. In this study, our aim was to determine the potential role of vitamin D in recurrent tonsillitis by measuring serum 25-OH vitamin D levels and determining the vitamin D receptor polymorphism among children with recurrent tonsillitis.

**Methods:**

Eighty-four children with recurrent tonsillitis and seventy-one healthy children aging between 2 and 10 years were enrolled in this study. Serum 25-OH vitamin D level was measured with ELISA and vitamin D receptor gene polymorphism (*Apa1, Taq 1, Fok1*) was determined by PCR. Serum 25-OH vitamin D level below 50 nmol/L was accepted as deficiency. The vitamin D receptor gene polymorphism in each group was compared.

**Results:**

The mean age was 5.6 ± 2.4 and 6.1 ± 2.7 years in study and control group, respectively. The average serum 25-OH vitamin D level was 142.7 ± 68.1 nmol/L in study group and 192.3 ± 56.1 nmol/L in control group. There was significant difference between the groups (p < 0.01). In study group, 4.7% (n = 4) of children had serum 25 OH vitamin D levels below 50 nmol/L. None of the children in control group had serum 25-OH vitamin D level below 50 nmol/L. There was no significant differences in vitamin D receptor gene polymorphisms between groups.

**Conclusion:**

Serum 25-OH vitamin D levels in recurrent tonsillitis group were lower than those in healthy children. But, there was no difference in the incidence of vitamin D receptor gene polymorphism between the two groups.

## Introduction

Tonsillopharyngitis is one of the leading causes of hospital visits during childhood and defined as the acute infection of tonsil and pharynx, characterized by erythema, exudation, ulceration or membrane [1]. It is a major health issue due to its frequent nature, causing labour loss and leading to unreasonable antibiotic use [2]. Besides viruses as the most frequent cause in etiology, the Group A Beta-haemolytic streptococci is the most commonly isolated bacteria [3]. As described by Paradise, at least 7 or more episodes of tonsillopharyngitis in a year or at least 5 episodes of acute tonsillitis in a year for two consequetive years or at least 3 or more episodes of acute tonsillitis in a year for three consequetive years is accepted as recurrent tonsillopharyngitis [4]. Although many factors are established for the cause of frequent tonsillopharyngitis, the exact cause has not been identified yet. The environmental conditions, the child’s immune system, mucosal characteristics effecting the bacterial biofilm formation on tonsillar tissue and the respond to the infections are of importance [5]. Antibiotics are not effective if bacterial biofilm occurred. Vitamin D has a preventive role for inhibition of bacterial biofilm. Even if the frequency of the disease during the winter is explained by environmental factors such as being indoor during winter, being deprived of sun light is also a point attracting attention. The most important factor provided by the sun is the production of vitamin D. Previous research suggests that vitamin D has an important role in immune system [6]. Vitamin D has an important role in maintaining of innate immunity. Assignation of vitamin D receptors (VDR) in many tissues has presented new ideas concerning the function of this vitamin. Vitamin D has numerous effects on different cells types of the immune system such as dendritic cells, B lymphocytes, T lymphocytes, NK cells [7-9]. Some polymorphism characteristics have been described regarding VDR gene affected by vitamin D. The frequently studied ones are Fok I, Bsm, Apa and Taq polymorphisms. Relation has been established between polymorphisms and some diseases [10,11].

In this study, our aim is to determine the role of the polymorphism characteristics of vitamin D and VDR receptors in children diagnosed with recurrent tonsillopharyngitis.

## Material and methods

### Study design

**Figure 1:**
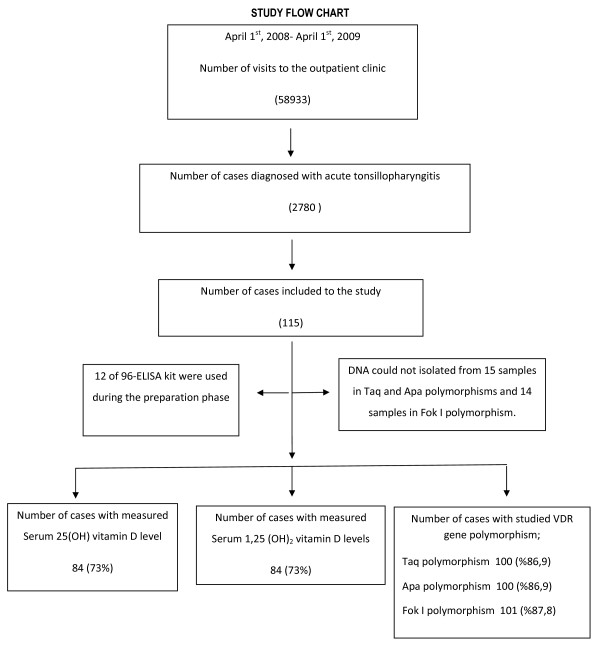
Flow chart of the study.

This clinical retrospective study was carried out among 242 children between 2 and 10 years of age performed in Istanbul University, Istanbul Medical Faculty, Department of Pediatrics, Outpatient Clinic of General Pediatrics between April 2008 and April 2009. During this period, the total number of access to the outpatient clinic was 58933. Four-point seven percent (2780) of these were due to acute tonsillopharyngitis. A hundred and fifteen of these met the eligilbility criteria and included to the study group (115/2780; 4.1%). A hundred and twenty-seven healthy children followed for different problems by outpatient clinic of the same institution included to the control group. This study was conducted mostly in autumn and winter seasons because of the increased frequency of tonsillopharyngitis during those seasons and seasonal variability of the vitamin D levels. Blood samples were collected from both groups and were studied in the same season. There were 115 cases who met the eligibility criteria of the study. The kit used for this study was designed to run 96 samples for both 25(OH) vitamin D and 1,25-(OH) vitamin D. Twelve out of 96 were used to measure 25(OH) vitamin D and 1,25-(OH) vitamin D levels during the preparation phase of the study. Thus, 25(OH) vitamin D and 1,25(OH)_2_ vitamin D levels were measured for 84 out of 115 subjects (Figure 1).

#### The eligibility criteria for participants

Inclusion criteria for the study group are as follows;

a) Having tonsillopharyngitis more than 7 times a year,

b) Having symptoms of tonsillopharyngitis developed within last 7 days,

c) No conditions requiring to be hospitalized,

d) Given parental consent,

e) No known chronic diseases,

f) Not having any supplementation of vitamin D during the last 3 month.

### Tonsillopharyngitis

Tonsillopharyngitis is diagnosed depending on the physical examination performed by a physician and subjects included to the the study regardless of the etiology, without performing any further microbiologic testing for the distinction of the viral or bacterial causes. The subjects that were diagnosed as tonsillopharyngitis more than 7 times in a year are considered as frequent tonsillopharyngitis. The frequency of tonsillopharyngitis in a year is investigated by reviewing the hospital records and by referring the feedback of parents if the patient is diagnosed with tonsillopharyngitis in a different institution or a prescription for tonsillopharyngitis was given by another physician previously.

### Measurement of serum vitamin D level and determination of vitamin D receptor (VDR) gene polymorphism

In this study serum 25(OH) and 1,25-(OH) vitamin D levels were measured by sandwich Enzyme Linked Immunosorbent Assay (ELISA) method. DNA of the subjects was isolated from the blood samples obtained and VDR’s Apa, Taq , Fok I polymorphisms were determined through primer series [12,13].

### Interpretation of serum vitamin D levels

Serum 25(OH) vitamin D level was interpreted as insufficient, sufficient, excess and intoxication if the blood level was between 50–80 nmol/L, 80–250 nmol/L, 250–325 nmol/L and above 325 nmol/L. Serum 25(OH) vitamin D level below 50 nmol/L was accepted as apperent deficiency (14) while the normal range of Serum 1,25-(OH)_2_ vitamin D was considered as 16–65 pg/ml [14].

### Factors affecting serum vitamin D level

Daily exposure to sunlight, gender, age, weight,, and the amount of time spent with outdoor activities and at seaside were investigated in both groups.

### Ethical committee approval and support

Istanbul University, Istanbul Shool of Medicine ethical committee approval was taken in January 2008 with the file numbered 2008/35 before the study was performed. This study was supported by Istanbul University Scientific Research Projects (Project No: 2006).

### Statistical analysis

In the evaluation of the qualitative and quantitative parameters, Student’s t-test, Chi-Square, Fisher’s exact chi-square, Pearson correlation analysis and logistic regression tests were utilized. Statistical analyses were made by SPSS 10.0 software program. The threshold for significance was p < 0.05.

## Results

The characteristics of the groups were as follows: 39.1% of study group (n: 45) and 44.9% of control group (n: 57) were girls (p = 0.36). The average age of study group was 5.6 ± 2.4 years and control group was 6.1 ± 2.7 years (p = 0.13). The average weight of study group was 21.3 ± 8.0 kg and of control group was 21.8 ± 8.7 kg (p = 0.72). The average height of study group was 113.8 ± 16.2 cm and of control group was 112.8 ± 19.3 cm (p = 0.74; Table 1). The average annual frequency of tonsillopharyngitis of study group was 15.3 ± 4.7 times and control group was 2.5 ± 0.9 times (p < 0.01).

**Table 1:** Main characteristics of the cases

	**GROUPS**		**P value**
	**Study Group**	**Control Group**	
	**(n:115)**	**(n:127)**	
Gender Girl; n (%)	45 (39.1)	57 (44.9)	0.36
Age (years)	5.6 ± 2.4	6.1 ± 2.7	0.13
Weight (kg)	21.3 ± 8.0	21.8 ± 8.7	0.72
Height (cm)	113.8 ± 16.2	112.8 ± 19.3	0.74
Annual tonsillopharyngitis frequency	15.3 ± 4.7	2.5 ± 0.9	<0.01

Study group consisted of the cases having frequent tonsillopharyngitis and control group consisted of healthy cases.

**Figure 2:**
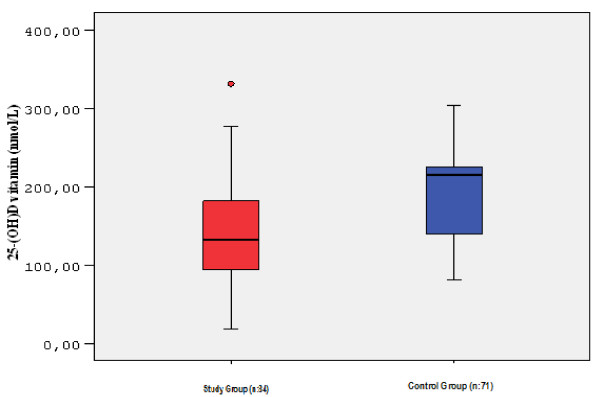
**Serum 25-(OH) vitamin D levels.** Serum 25-OH vitamin D levels were measured in 84 cases in study group and in 71 cases in control group.

In study group, 25(OH) vitamin D level was studied in 84 of 115 cases (84/115; 73%). The average serum 25(OH) vitamin D level was 142.7 ± 68.1 nmol/L. The lowest serum 25(OH) vitamin D level was measured as 18 nmol/L and the highest level was 331 nmol/L. Serum 25 (OH) vitamin D level of 4 cases (4/84; 4.7%) was below 50 nmol/L, which was considered as the cut off for vitamin D deficiency. The values of these 4 cases were measured as 18, 24, 39, 49 nmol/L. In one case, serum 25(OH) vitamin D level was found above 325 nmol/L considered as above the intoxication limit. In control group, 25(OH) vitamin D level was studied in 71 of 127 cases (71/127; 55.9%). The average serum 25(OH) vitamin D level was 192.3 ± 56.1 nmol/L. The lowest level was measured as 81 nmol/L and the highest level was 303.7 nmol/L. No cases were found at deficiency or intoxication limit in control group. The average serum 25(OH) vitamin D level of study group was significantly lower than that of control group (p < 0.01) (Table 2 and Figure 2).

**Table 2:** Serum levels of 25-OH vitamin D levels were measured in 84 patients in study group and 71 patients in control group

	**Study Group**	**Control Group**	**P value**
Serum 25(OH) vitamin D level (nmol/L)	142.7 ± 68.1	192.3 ± 56.1	<0.01
Serum 1,25-(OH)_2_ vitamin D level (pg/mL)	35.8 ± 16.4	28.4 ± 14.1	<0.01
Number of patients with serum 25-OH vitamin D level below <50 nmol/L	4 (4/84; 4,5%)	0	0.04

Serum 1,25 (OH)_2_ vitamin D was measured in 84 patients in study group and 79 patients in control group.

In study group, serum 1,25-(OH)_2_ vitamin D level was also studied in 84 of 115 cases (73%). The average serum 1,25-(OH)_2_ vitamin D level was 35.8 ± 16.4 pg/ml. The lowest level was measured as 12.2 pg/ml and the highest level was 81.5 pg/ml. In 3 cases, 1,25-(OH)_2_ vitamin D level was below normal limit (Values: 12.2, 13.5, 13.7 pg/ml). In 6 cases, 1,25-(OH)_2_ vitamin D level was above normal limit (Values: 66.2, 78.8, 80.1, 80.4, 80.5, 81.5 pg/ml). In control group, serum 1,25-(OH)_2_ vitamin D level was studied in 79 of 127 cases (62.2%). The average serum 1,25-(OH)_2_ vitamin D level was 28.6 ± 14.1 pg/ml. The lowest level was measured as 7.4 pg/ml and the highest level was 66.2 pg/ml. In 10 cases from control group, 1,25-(OH)_2_ vitamin D level was below normal limit (Values: 7.4, 7.9, 8.1, 8.2, 10.4, 10.8, 10.9, 11.5, 13, 14.3 pg/ml). In one case, 1,25-(OH)_2_ vitamin D level was above normal limit (66.2 pg/ml). The average 1,25-(OH)_2_ vitamin D level of study group was significantly higher than that of control group (p < 0.01).

Regarding *Taq, Apa* and *Fok I* polymorphisms in VDR receptor gene, no statistically significant difference was found between study and control groups (p values: 0.99, 0.14, 0.55, respectively, Table 3).

**Table 3:** Vitamin D receptor gene polymorphism

**VDR**	**Gene Polymorphisms**	**Study Group**	**Control Group**	**P value**
		**(n:100) (n; (%))**	**(n:104) (n; (%))**	
Taq	TT	49 (49)	50 (48.5)	0.99
	Tt	40 (40)	42 (40.8)	
	tt	11 (11)	11 (10.7)	
Apa	AA	39 (39)	43 (42.6)	0.14
	Aa	53 (53)	58 (57.4)	
	aa	8 (8)	0 (0)	
Fok I	FF	46 (45.5)	54 (53.5)	0.55
	Ff	52 (51.5)	41 (40.6)	
	ff	3 (3)	6 (5.9)	

VDR gene polymorphism was analyzed in 100 cases for study group and in 104 cases for control group.

In multivariate analysis, the most efficient parameters on annual disease frequency were serum 25(OH) vitamin D and 1,25(OH)_2_ vitamin D levels; the annual disease frequency increases with “Ff” polymorphism, whereas decreases with “ff” polymorphism.

## Discussion

The existence of VDRs in immune system cells and different modulatory effects upon the stimulation of these receptors have brought forward the relation of vitamin D and immune system [5,6]. The best parameter for the evaluation of vitamin D level and vitamin D pool in the body is serum 25 (OH) vitamin D levels, whose half life of is about 20 days [13-15]. Since the half life of the biologically active form, 1,25-(OH)_2_ vitamin D is as short as 3 to 6 hours and the circulating blood level is very low compared to 25(OH) vitamin D, its use is not preferred for the evaluation of serum vitamin D levels [16]. In our research, we studied the level of 25(OH) vitamin D which gives information on body vitamin D storage and also the level of biologically active form, 1,25-(OH)_2_ vitamin D together. Due to the ease of the technique, serum 25(OH) vitamin D and 1,25-(OH)_2_ vitamin D levels were measured by ELISA method. Vitamin D is effective on all other systems apart from its important role in bone and mineral (calcium, phosphorus) metabolisms. Since our study has been carried out on children having frequent tonsillopharyngitis that is not associated with bone-mineral system; serum calcium, phosphorous, alkaline phosphatase and parathormone levels were not measured.

The human VDR is polymorphic [16]. Recently, a relation was established between VDR gene polymorphisms and predisposition to some diseases [10]. Presence of the t allele of the TaqI VDR receptor polymorphism is associated with increased anti-infective action against tuberculosis [17,18]. In contrast, presence of the f allele of the FokI VDR polymorphism is associated with reduction in anti-infective action against tuberculosis [17,19]. The significance of VDR polymorphisms on vitamin D production and activity is a developing field. We tried to establish a relation between VDR gene polymorphism and frequent tonsillopharyngitis in children. In our study, we observed that annual disease frequency increases in the patients with “Ff” polymorphism, whereas it decreases in those ones with “ff” polymorphisms. Our findings were in contrast with the literature demonstrating negative correlation between VDR ff allele polymorphism and recurrent tonsillopharyngitis [20].

Although the average serum 25-(OH) vitamin D levels were in the normal range in both groups, it was significantly lower in the children with frequent tonsillopharyngitis (p < 0.01). In our study, we tried to determine the serum 25-(OH) vitamin D level that effectively prevents tonsillopharyngitis. However, because of the limited number of cases in our study, we were not able to make this assessment. But some preliminary data showed that serum 25-OH vitamin D levels are lower in patients with recurrent tonsillopharyngitis [21]. Two randomized controlled trails reported that, vitamin D supplementation is effective for prevention of upper respiratory tract infections [22,23]. To determine the minimal serum vitamin D level necessary to protect against upper respiratory tract infections, more cohort studies with a larger number of cases are needed. The difference of serum 1,25-(OH)_2_ vitamin D levels between the groups may be because of the short half-life of serum 1,25-(OH)_2_ vitamin D and the difference in study timing of the samples at the laboratory.

Aydin and colleagues [24] conducted a prospective research. Aydin and colleagues checked the serum 25- (OH) vitamin D levels in tonsillectomy patients with recurrent tonsillitis, whilst our group checked the serum 25- (OH) vitamin D and 1.25 (OH) 2 vitamin D levels of children with frequent tonsillopharyngitis. Whilst both studies into serum 25- (OH) vitamin D levels were within the normal range, Aydin and colleagues found no statistical differences with the control group. Our study did show a statistical difference.

In our study, the children who had recurrent tonsillopharyngitis during the one-year period were determined and their serum vitamin D levels were studied. Since vitamin D is affected by external factors and displays seasonal differences in particular, studying serum vitamin D levels by means of taking serum samples in both summer and winter would have been ideal. Evaluating the nutritional characteristics of the children would make the study more reliable.

In conclusion, based on this study, low serum vitamin D level can be a risk factor for recurrent tonsillopharyngitis. In children having recurrent tonsillopharyngitis, serum 25(OH) vitamin levels were lower than those of healthy children. But, there was no difference in vitamin D receptor gene polymorphism. In the future, giving vitamin D support to the children with recurrent tonsillopharyngitis, then measuring their serum vitamin D levels and observing the decrease in annual disease frequency after the supportive treatment would exactly explain the relation between vitamin D and having recurrent tonsillopharyngitis.

## Competing interests

The authors declare that they have no competing interests.

## Authors’ contributions

IY, EU, AK and SA carried out the clinic studies. UZ, BT and CC participated in measurements of the levels of vitamin D. ST carried out the statistical analysis. All authors participated to drafted the manuscript. All authors read and approved the final manuscript.
